# Achieving the Impossible

**DOI:** 10.31486/toj.26.5059

**Published:** 2026

**Authors:** Ronald G. Amedee

**Affiliations:** Director of Clinical School and Professor, The University of Queensland Medical School, Ochsner Clinical School, New Orleans, LA; Editor-in-Chief, *Ochsner Journal*

*It always seems impossible until it's done.*

–*Nelson Mandela*

The Spring 2026 issue of *Ochsner Journal* contains one editorial, two original research articles, one innovative program, and seven case reports and clinical observations that serve as the principal elements for this edition. This issue also contains a section entitled “Additions” which includes six selected abstracts from the inaugural University of Queensland-Ochsner Doctor of Medicine Program Year 3 Scholarly Project.

The editorial that begins the issue focuses on the barriers to access for a drug approved in 2023 to treat postpartum depression. Authors are University of Queensland Medical School-Ochsner Clinical School student Simmers and faculty member Galarneau.

The first original research article is from Luminis Health Anne Arundel Medical Center in Annapolis, Maryland, authored by Baz, Paxton, Turcotte, Milburn, Woodward, and Jackson entitled “Management and Outcomes of Complications Related to BioZorb Use in Breast-Conserving Surgery: A Retrospective Single-Center Case Series Analysis.” This paper is followed by “Trends in Obesity, Overweight, and Attempted Weight Loss Among United States High School Students” authored by Yang, Krill, Llorens, Kunz-Lomelin, Hennekens, and Kitsantas from Charles E. Schmidt College of Medicine, Florida Atlantic University, Boca Raton, Florida.

The innovative program submission is entitled “Medical Student Screening of Social Determinants of Health in Urogynecology Surgical Patients” authored by University of Queensland Medical School-Ochsner Clinical School students and faculty Anderson, Biggs, Mishra, Jordan, and Knoepp.

The case reports and clinical observations section begins with “Thigh Compartment Syndrome Following Physician-Modified Fenestrated Endograft Aneurysm Repair” authored by Crowley, Bigham, Hurley, Parkerson, and Dunbar from Ochsner Orthopedics and Vascular Surgery. This case is followed by “Soft Tissue Surgical Technique for Obligate Dislocation of the Patella” authored by Minkowitz, Kasarla, Spingarn, Matalon, Avallone, and Busch, with most authors from Morristown Medical Center in Morristown, New Jersey. Next, Koos, Shariati, and Anand from Ochsner Internal Medicine and Cardiology present “Immune Checkpoint Inhibitor-Induced Myocarditis: A Late Presentation.” Buitrago, Spring, and Conway from Ridley-Tree Cancer Center in Santa Barbara, California, detail a case of “Isolated Herniation of Gallbladder Through Diaphragmatic Defect Following Hepatic Microwave Ablation,” followed by “The Deceptive Nature of Pneumatosis Intestinalis: From Spontaneous Resolution to Bowel Ischemia” authored by Baz, Bussey, and Wormuth, also from Luminis Health Anne Arundel Medical Center, Annapolis, Maryland. This case is followed by “Stener-Like Lesion of the Radial Collateral Ligament of the Thumb Metacarpophalangeal Joint” authored by an Ochsner Clinical School medical student and Ochsner Orthopedics faculty. Rounding out this section is “Phlegmasia Cerulea Dolens Secondary to Iliac Vein Compression From Posttransplant Hematoma Despite Anticoagulation” by Lim, Parkerson, and Barr, from an Ochsner Clinical School student and Ochsner Surgery/Transplant Surgery team of authors.

Many medical school faculty from Brisbane, Australia, and New Orleans, Louisiana, doubted in 2024 that a longitudinally integrated curriculum element could be delivered in the refreshed medical school curriculum of year 3. At the time, this curricular addition was deemed to be impossible, but it was introduced as a year-long scholarly project in January 2025 and successfully completed in October the same year. In the final “Additions” section of this Spring 2026 issue, please find six selected abstracts from teams completing their projects exclusively in New Orleans. I encourage you to read the abstracts and want to thank faculty supervisors for their exceptional involvement in these projects and the medical students who learned extensively through this effort.

